# COVID-19 Outbreak in a Hemodialysis Center Using Universal Masking and Eye Protection

**DOI:** 10.1017/ash.2021.96

**Published:** 2021-07-29

**Authors:** Costi Sifri, Joan Buckner, Cady Shelton

## Abstract

**Background:** Up to half of all new SARS-CoV-2 infections are acquired from presymptomatic or asymptomatic individuals. Hemodialysis patients and healthcare providers (HCPs) may be at increased risk for COVID-19 due to the need for extended close contact. Universal masking and eye protection are strategies used to reduce SARS-CoV-2 exposure, particularly from presymptomatic or asymptomatic individuals. We describe an outbreak of COVID-19 in an outpatient hemodialysis center despite universal masking and universal HCP eye protection. **Methods:** An COVID-19 outbreak investigation was performed in a hemodialysis center where universal masking was in use by all HCPs and patients and universal eye protection (goggles, safety glasses) was in use by all HCPs when directly encountering patients. After a cluster of cases was identified in early November 2020, all patients and HCPs were tested for SARS-CoV-2 by real-time reverse transcription polymerase chain reaction (RT-PCR) when symptomatic and weekly until the conclusion of the outbreak. **Results:** From November 12, 2020, through December 7, 2020, 14 (23.3%) of 60 patients and 9 (28.1%) of 32 HCPs tested positive for SARS-CoV-2 by RT-PCR (Figure 1). The median ages of the patients and HCPs were 64 years (range, 42–87) and 42 years (range, 29–68), respectively. Also, 5 (5.4%) individuals (3 patients and 2 HCPs) were asymptomatic at the time of testing. Furthermore, 7 (7.6%) individuals (5 patients and 2 HPCs) were hospitalized; 2 patients and no HCPs died. No lapses in universal masking or, for HCPs, eye protection prior to or during the outbreak were identified during the investigation. All HCPs and patients wore medical-grade face masks that were discarded at the end of the day; HCPs wore safety glasses or goggles during patient interactions. Although audits of face mask and eye protection compliance were not performed, independent interviews supported high HCP and patient adherence prior to the outbreak. Neither the staff nor patients shared meals at or outside the hemodialysis center. Most patients and HCPs shared the same hemodialysis shift, suggesting the presence of an index case; however, a source case could not be identified despite an extensive investigation. **Conclusions:** Universal masking and eye protection have been shown to reduce transmission of SARS-CoV-2 from presymptomatic or asymptomatic individuals in the healthcare setting. This report suggests that such measures cannot prevent all outbreaks. We speculate that certain factors associated with hemodialysis care, such as prolonged close patient–HCP contact, may have facilitated this outbreak. Whether nonadherence to universal masking and eye protection or failure of empiric droplet precautions contributed to this outbreak remains unknown.

**Funding:** No

**Disclosures:** None

